# Peroxidase(s) in Environment Protection

**DOI:** 10.1155/2013/714639

**Published:** 2013-12-24

**Authors:** Neelam Bansal, Shamsher S. Kanwar

**Affiliations:** Department of Biotechnology, Himachal Pradesh University, Shimla 171 005, India

## Abstract

Industrial discharges of untreated effluents into water bodies and emissions into air have deteriorated the quality of water and air, respectively. The huge amount of pollutants derived from industrial activities represents a threat for the environment and ecologic equilibrium. Phenols and halogenated phenols, polycyclic aromatic hydrocarbons (PAH), endocrine disruptive chemicals (EDC), pesticides, dioxins, polychlorinated biphenyls (PCB), industrial dyes, and other xenobiotics are among the most important pollutants. Peroxidases are enzymes that are able to transform a variety of compounds following a free radical mechanism, thereby yielding oxidized or polymerized products. The peroxidase transformation of these pollutants is accompanied by a reduction in their toxicity, due to loss of biological activity, reduction in the bioavailability, or the removal from aqueous phase, especially when the pollutant is found in water. The review describes the sources of peroxidases, the reactions catalyzed by them, and their applications in the management of pollutants in the environment.

## 1. Introduction

Two unpredicted challenges for human being are energy and environment. Functioning of society as a whole and its future progress are dependent on the availability of new and renewable sources of energy and on the capacity to change polluting productive processes for new environment friendly processes. Together, these developments have led to a growing awareness of the central importance for the environmental sciences as humankind attempts to transition to a more sustainable relationship with the Earth and its natural resources [1]. Peroxidases have potential to decrease environmental pollution by bioremediation of waste water containing phenols, cresols, and chlorinated phenols, for biopulping and decolourization of synthetic textile azo-dyes. Peroxidases (EC 1.11.1.7) are oxidoreductases that catalyse the reduction of peroxides, such as hydrogen peroxide (H_2_O_2_) and the oxidation of a variety of organic and inorganic compounds [2, 3]. Specifically, peroxidase activity involves donating electrons that bind to other substrates such as ferricyanides and ascorbate, in order to break them into harmless components.

Peroxidases have potential for bioremediation of waste water contaminated with phenols, cresols, and chlorinated phenols, for biopulping [4] biobleaching in paper industry, textile-dye degradation, and removal of peroxide from materials such as food stuffs and industrial wastes. Process water from textile mills often features a strong coloration due to presence of rhodamine dyes which are resistant to conventional bleaching treatment and can be degraded by peroxidase [5]. The unique ability of white rot fungi to degrade lignin is largely attributable to the nonspecific free radical mediated oxidizing reactions carried out by their extracellular peroxidases [6]. Peroxidase oxidizes dimethoxybenzene, lignin dimers, phenols, amines, dyes, and aromatic alcohols in the absence of Mn(II); peroxidase oxidizes phenolic and nonphenolic substrates. Yet another peroxidase, designated dye-decolorizing peroxidase from *Agaricus* type fungi, was reported to catalyze the oxidation of dyes and phenolic compounds [7]. Peroxidases from different sources are relatively nonspecific and provide white rot fungi the unique ability to degrade abroad array of environmental pollutants such as dioxins, polychlorinated biphenyls, petroleum hydrocarbons, munitions wastes (such as trinitrotoluene), industrial dye effluents, herbicides, and pesticides [8].

## 2. Sources of Peroxidase

**Figure 1:**
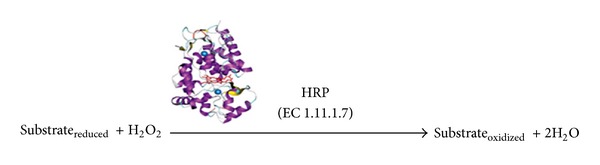
A general reaction catalyzed by HRP.

Peroxidases (EC 1.11.1.7) are widely distributed in nature. These enzymes are produced by a variety of sources including plants, animals, and microbes. Peroxidases produced from microbial sources such as bacteria (*Bacillus sphaericus*, *Bacillus subtilis*, *Pseudomonas* sp., *Citrobacter* sp.), Cyanobacteria (*Anabaena* sp.), fungi (*Candida krusei*, *Coprinopsis cinerea*, *Phanerochaete chrysosporium*), actinomycetes (*Streptomyces* sp., *Thermobifida fusca*), and yeast are used in decomposition of pollutants, production of animal feedstock, and raw materials for the chemical, agricultural, paper industries, textile dye degradation, paper-pulp industry for lignin degradation, dye decolorization, sewage treatment, and also as biosensors. Many plant sources for peroxidases production have been reported such as horseradish, papaya (*Carica papaya*), banana (*Musa paradisiacal*), and bare (*Acorus calamus*). Peroxidase obtained from horseradish (HRP) is extensively used in diagnostic kits, in ELISA for labeling an antibody, synthesis of various aromatic chemicals, and removal of peroxides from materials such as foodstuff and industrial wastes (Figure 1).

## 3. Characteristics of Peroxidase(s)

Peroxidases are oxidoreductases that catalyse a variety of reactions such as reduction of peroxides such as hydrogen peroxide and oxidation of a variety of organic and inorganic compounds. These are heme proteins and contain iron (III) protoporphyrin IX as the prosthetic group. They have a molecular weight ranging from 30 to 150 kDa. The term peroxidase represents a group of specific enzymes, such as NADH peroxidase (EC 1.11.1.1), glutathione peroxidase (EC 1.11.1.9), and iodine peroxidase (EC 1.11.1.8), as well as a variety of nonspecific enzymes that are simply known as peroxidases.

## 4. Applications and Peroxidase Biocatalysis in Management of Environmental Pollutants

### 4.1. Decolorization of Synthetic Dyes

Dye wastes represent one of the most problematic groups of pollutants considered as xenobiotics that are not easily biodegradable [9]. These dyes are mostly used in textile dyeing, paper printing, colour photography, and as additive in petroleum products. When these synthetic dyes are discharged into industrial effluents they cause environmental pollution. Textile industries play a vital role in the economic increases in India. Water is one of the major products of nature used enormously by human beings, and it is not unnatural that any growing community generates enormous waste water or sewage [10]. To achieve the biodegradation of environmentally hazardous compounds, white rot fungi appear as a valuable alternative. The capability of oxidation is based on the ability of white rot fungi to produce oxidative enzymes such as laccase, manganese peroxidase, and lignin peroxidase [11]. These oxidases and peroxidases have been reported as excellent oxidant agents to degrade dyes [12].

Several bacterial peroxidases have been used for decolorization of synthetic textile dyes. Removal of chromate Cr (VI) and azo dye Acid Orange 7 (AO7) using *Brevibacterium casei* under nutrient-limiting conditions has been studied. AO7 was used as an electron donor by the reduction enzyme of *Brevibacterium casei* for the reduction of Cr (VI). The reduced chromate Cr (III) complexed with the oxidized AO7 formed a purple intermediate [13]. Decolorization of different azo dyes by *Phanerochaete chrysosporium* RP 78 under optimized conditions was studied [14] by reaction mechanism *via* azo dye. Peroxidase was produced under aerobic conditions as a secondary metabolite in the stationary phase. *Bacillus* sp. VUS isolated from textile effluent contaminated soil showed capability for degrading a variety of dyes [15]. The production of ligninolytic peroxidases directly oxidizing aromatic compounds has been described in fungi [16]. Other peroxidases were detected in microorganisms responsible for the biodegradation of industrial dyes together with lignin peroxidase [17]. An edible macroscopic fungi *Pleurotus ostreatus* produced an extracellular peroxidase that can decolorize remazol brilliant blue and other structurally different groups including triarylmethane, heterocyclic azo, and polymeric dyes. Bromophenol blue was decolorized best (98%), while methylene blue and toluidine blue O were least decolorized 10% [18]. HRP was found to degrade industrially important azo dyes such as remazol blue. This dye contains at least one aromatic group in its structure making it a possible substrate of HRP [19]. The dyeing and bleaching unit's contaminants seeping into the ground have polluted the ground water rendering it unsuitable for consumption (Table 1).

### 4.2. Bioremediation of Waste Water: Removal of Phenolic Contaminants and Related Compounds

Industrial pollution has been a major factor causing the degradation of the environment around us, affecting the water we use; its quality and human health is directly related issues. Improved quality and increased quantity of water would bring forth health benefits. Safe water eliminates the infective agents associated with water borne diseases; availability of greater quantity of water can improve health by allowing improved personal hygiene. Water pollution caused industrial waste products to release into lakes, rivers, and other water bodies that make marine life no longer hospitable. Peroxidases have been applied to the bioremediation of waste waters contaminated with phenols, cresols, and chlorinated phenols [2, 9]. Aromatic compounds including phenols and aromatic amines constitute one of the major classes of pollutants. They are found in the waste waters of a wide variety of industries, including coal conversion, petroleum refining, resins and plastics, wood preservation, metal coating, dyes and other chemicals, textiles, mining and dressing, and pulp and paper industries [34]. Phenols and halogenated phenols present in textile industries processed water are known to be toxic and also some of them are hazardous carcinogens that can accumulate in the food chain [5].

Peroxidases comprise an important class of enzymes able to catalyze the oxidative coupling reactions of a broad range of phenolic compounds [35]. Lignin peroxidase from *Phanerochaete chrysosporium*, HRP, myeloperoxidase, lactoperoxidase, microperoxidase-8, a versatile peroxidase from *Bjerkandera adusta*, and chloroperoxidase from *Caldariomyces fumago* [36] were able to transform pentachlorophenol totetrachloro-1,4-benzoquinone by an oxidative dehalogenation in the presence of H_2_O_2_. An extracellular manganese peroxidase produced by *P. chrysosporium*, *P. sordida*, *C. subvermispora*, *P. radiata*, *D. squalens*, and *P. rivulosu*. Two electron oxidation of that extracellular peroxidase by H_2_O_2_ yields Compound I which undergoes two consecutive one-electron reduction steps by oxidizing Mn^2+^ into Mn^3+^ that in turn oxidizes phenolic compounds [37]. Many toxic aromatic and aliphatic compounds occur in waste water of a number of industries. Among these, phenol is the most common aromatic pollutant and is also found in contaminated drinking water. Phenol can be toxic when present at an elevated level and is known to be carcinogenic. It has an effect on health even at low concentration. A laboratory phenol was treated with turnip root enzyme (peroxidase) extract in the presence of H_2_O_2_ as an oxidant to form corresponding free radicals. Free radicals polymerize to form substances that are less soluble in water. The precipitates were removed by centrifugation and residual phenol was estimated [38]. The results showed that turnip root enzyme extract degraded phenol more efficiently. Another versatile peroxidase produced by *P. eryngii* and *P. ostreatus* oxidized Mn^2+^ into Mn^3+^ similar to the action of MnP, and also high redox potential aromatic compounds, as LiP does, had broad specificity and oxidized nonphenolic compounds [39].

#### 4.2.1. Mechanism of HRP-H_2_O_2_-Phenol Reaction

Horseradish peroxidase undergoes a cyclic reaction when reacting with phenolic substrates. This sequence is summarized in the following reactions:

(1) $$\begin{array}{c} \text{E}+{\text{H}}_{2}{\text{O}}_{2}\to \text{Ei}+{\text{H}}_{2}\text{O}, \end{array}$$

(2) $$\begin{array}{c} \text{Ei}+\text{Ph}{\text{OH}}^{\prime }\to \text{Eii}+\text{PhO}, \end{array}$$

(3) $$\begin{array}{c} \text{Eii}+\text{Ph}{\text{OH}}^{\prime \prime }\to \text{E}+\text{PhO}+{\text{H}}_{2}\text{O}. \end{array}$$

The enzyme starts in its native form (E) and is oxidized by H_2_O_2_ to form an active intermediate compound known as compound 1 (Ei). Compound 1 oxidizes one molecule of phenol (PhOH) to form a phenol free radical (PhO) and become compound II (Eii). Compound II oxidizes a second phenol molecule to produce another phenol free radical and complete the cycle by returning to its native form E. The free radicals polymerize and form insoluble compounds which precipitate from solution [40]. The polymerization reaction is illustrated in

(4) $$\begin{array}{c} \text{PhO}+\text{PhO}\to \text{Polymer}\;\text{of}\;\text{aromatic}\;\text{compounds}. \end{array}$$

**Figure 2:**
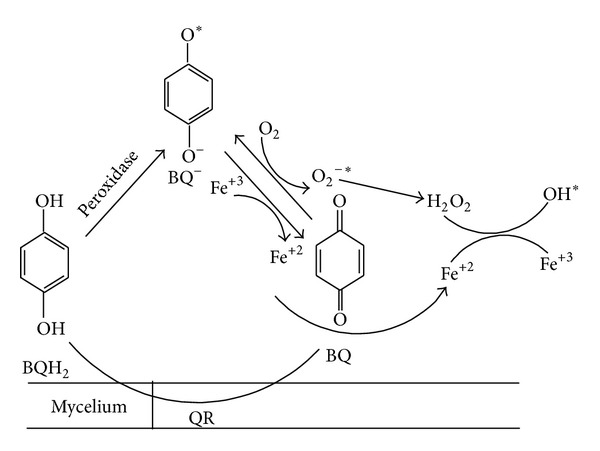
Reaction scheme involved in the production of hydroxyl radical by white rot fungi via quinone redox cycling [33]. 1,4-benzoquinone (BQ) is reduced by quinone reductase (QR) producing hydroquinone (BQH_2_), which is oxidized by any of the lignin modifying enzymes to semiquinones (BQ^−^). The production of superoxide anion radicals (O_2_^−^) by BQ^−^ autoxidation is mainly catalyzed by Fe^3+^ that is reduced to Fe^2+^. Fenton's reagent formation is accomplished by O_2_-dismutation to H_2_O_2_.

Yet another peroxidase, designated dye-decolorizing peroxidase (EC 1 : 1 : 1 : *x*) from *Agaricus* type fungi, was reported to catalyze the oxidation of dyes and phenolic compounds [7] (Figure 2).

### 4.3. Removal of Endocrine Disruptive Chemicals (EDCs)

Several classes of oxidative enzymes have shown promise for efficient removal of EDCs that are resistant to conventional waste water treatments. Although the kinetics of reactions between individual EDCs and selected oxidative enzymes such as HRP are well documented in the literature, there has been little investigation of reactions with EDC mixtures [41]. EDCs are a group of compounds that due to their chemical structure are able to act as agonists or antagonists of hormones. They can disturb the synthesis, secretion, transport, binding, action, and elimination of the endogenous hormones, which are responsible for maintaining homeostasis, reproduction, development, and integrity in living organisms and their progeny [42]. They are widely dispersed in the environment but are mainly found in waste water effluents. Several works reported the EDC oxidation by manganese peroxidase. Using 10 U/mL of manganese peroxidase from *Pleurotus ostreatus*, 0.4 mM bisphenol was eliminated in 1 h [43]. Peroxidases are also helpful in removal or degradation of other potent environmental pollutants such as chloroanilines and polycyclic aromatic hydrocarbons [44].

### 4.4. Degradation of Polychlorinated Biphenyls (PAHs) Pesticides

Pesticides include a broad range of substances most commonly used to control insects, weeds, and fungi. Pesticide exposure in human is associated with chronic health problems or health symptoms such as respiratory problems, memory disorders, dermatologic conditions, cancer, depression, neurologic deficits, miscarriages, and birth defects [45]. Biological decomposition of pesticides is the most important and effective way to remove these compounds from the environment. Microorganisms have the ability to interact, both chemically and physically, with substances leading to structural changes or complete degradation of the target molecule [46].

Peroxidases extracted from some fungal species have great potential to transform several pesticides into harmless form(s). Transformation of organophosphorus pesticides by white rot fungi has been studied [47], and transformation of several organophosphorus pesticides by the chloroperoxidase from *Caldariomyces fumago* has been reported. PAHs are composed of two or more fused aromatic rings and are components of crude oil, creosote, and coal [48]. Most of the contamination by PAHs had originated from the extensive use of fossil fuels as energy sources. Peroxidases and phenol oxidases can act on specific PAH's by transforming them to less toxic or products easier to degrade. PAHs are oxidized by peroxidases such as lignin peroxidase [49] and manganese peroxidase [50]. In spite of their versatility and potential use in environmental processes, peroxidases are not applied at large scale yet. Diverse challenges, such as stability, redox potential, and the production of large amounts, should be addressed in order to apply peroxidases in the pollutant transformation [51]. Peroxidases extracted from some fungal species have great potential to transform several pesticides into harmless form(s). In spite of their versatility and potential use in environmental processes, peroxidases are not applied at large scale yet. Diverse challenges, such as stability, redox potential, and the production of large amounts, should be addressed in order to apply peroxidases in the pollutant transformation.

### 4.5. Degradation of Chlorinated Alkanes and Alkenes

Contamination of soils and aquifers by the aliphatic halocarbons trichloroethylene (TCE) and perchloroethylene (PCE) widely used as degreasing solvents is a serious environmental pollution problem. TCE is subject to *in vitro* reductive dehalogenation catalyzed by LiP of *P. chrysosporium* in the presence of tertiary alcohol, H_2_O_2_, and EDTA (or oxalate) leading to the production of the corresponding reduced chlorinated radicals [52]. One strain of bacterium IM-4 capable of degrading imazethapyr (IMZT) was isolated from the IMZT-contaminated soil. This strain also showed the capability to degrade other imidazolinone herbicides such as imazapyr, imazapic, and imazamox [53]. Extracellular hydroxyl radicals produced by *T. versicolor*, via quinone redox cycling, were also shown to catalyze degradation of PCE and TCE [1]. TCE is mineralized by *P. chrysosporium* cultures grown aerobically. These investigators proposed that TCE is subject to *in vitro* reductive dehalogenation catalyzed by LiP of *P. chrysosporium* in the presence of tertiary alcohol, H_2_O_2_, and EDTA (or oxalate) leading to the production of the corresponding reduced chlorinated radicals [8].

### 4.6. Degradation of Phenoxy Alkanoic and Triazineherbicides

The most commonly used broad leaf herbicides around the world are 2,4-dichlorophenoxyacetic acid (2,4-D) and 2,4,5-trichlorophenoxyaceticacid (2,4,5-T). 2,4-D and perhaps 2,4,5-T are a component of Agent Orange that was widely used as a defoliant. 2,4-D is quite susceptible to bacterial degradation and generally does not persist for long in the environment. On other hand 2,4,5-T is relatively more resistant to microbial degradation and tends to persist in the environment. It has been blamed for serious illnesses in many veterans of Vietnam War, where they got exposed to Agent Orange that was used as a defoliant. These were also reported to be mutagenic agents and thus very toxic to humans. Ligninolytic peroxidases of *P. chrysosporium* and *Dichomitus qualens* were involved in the degradation of chlorinated phenolic intermediates of 2,4-D and 2,4,5-T. These results were based on the increased degradation of ring-labeled and side chain-labeled 2,4,5-T and 2,4-D by *D. Squalens* on addition of Mn^2+^ (a known inducer of MnP) to the medium and on increased degradation by *P. chrysosporium* in nitrogen-limited medium (in which production of both LiP and MnP is induced). Atrazine is a commonly used triazine herbicide and is degraded by a number of white rot fungi produced laccases and peroxidase [54].

### 4.7. Degradation of Chlorinated Dioxins

Polychlorinated dibenzodioxins (PCDDs) are a group of highly toxic environmental pollutants that are confirmed human carcinogens and tend to bioaccumulate in humans and animals due to their lipophilic properties. Polychlorinated dibenzodioxins (PCDD) and polychlorinated dibenzofurans (PCDF) have been shown to be degraded by several species of white rot fungi [55] suggesting the possible involvement of LiP and MnP. A fungus *P. sordida* produced MnP but no LiP; and crude MnP showed degradation of the dioxins.

### 4.8. Degradation of Chlorinated Insecticides

Lindane (c isomer of hexachlorocyclohexane) was a widely used pesticide in the past, and an estimated 600,000 tons of lindane were produced globally between the year 1950 and 2000. There is a global ban on the use of lindane now because of its environmental persistence as a pollutant. *P. chrysosporium* cultured under ligninolytic conditions was reported to partially mineralize lindane in liquid cultures and in a corncob-amended soils inoculated with *P. chrysosporium* [56] but lindane degradation was not observed *in vitro* using purified LiP and MnP from *P. chrysosporium* [57]. DDT (1,1,1-trichloro-2,2-bis [4-chlorophenyl] ethane), the first of the chlorinated organic insecticides, was used quite heavily after World War II. High levels of DDT found in agricultural soils are of deep concern, because they present serious threats to food security and human health. The white rot fungi *P. chrysosporium*, *P. ostreatus*, *T. versicolor*, and *Phellinus weirii* have been shown to mineralize DDT [58].

### 4.9. Peroxidase as Biosensors

Biosensors have been defined as analytical devices which tightly combine biorecognition elements with physical transducers for detection of the target compound. Several examples of biosensors developed for relevant environmental pollutants. Biosensors can be useful, for example, for the continuous monitoring of a contaminated area [59]. They may also present advantageous analytical features, such as high specificity and sensitivity (inherent in the particular biological recognition bioassay. H_2_O_2_ is considered the mediator of the biochemistry of cellular pathology and maybe involved in the etiology of aging and progressive neurodegenerative diseases such as Parkinson's disease. Due to its crucial role in neurochemistry, determination of the concentration of H_2_O_2_ has been a considerable interesting research field. Electrochemical methods based on peroxidase biosensors have proved to be significantly advantageous to the biosciences due to their direct real-time measurements and capability for practical applications [60]. A novel third generation biosensor for hydrogen peroxide was constructed by cross-linking HRP onto an electrode modified with multiwall carbon nanotubes [61]. At the same time, biosensors offer the possibility of determining not only specific chemicals but also their biological effects, such as toxicity, cytotoxicity, genotoxicity, or endocrine disrupting effects, that is, relevant information that in some occasions is more meaningful than the chemical composition. Enzymatic biosensors are based on the selective inhibition of specific enzymes by different classes of compounds, with the decrease in activity of the immobilized enzyme in the presence of the target analyte as the parameter that is frequently used for quantification.

A novel myoglobin-based electrochemical biosensor based on a nanocomposite prepared from multiwalled carbon nanotubes that were coated with ceria nanoparticles has been developed [62]. Another application of whole-cell biosensors is the determination of the biological oxygen demand (BOD). Pesticides (herbicides, fungicides, and insecticides) are widely used in the agriculture and industry around the world due to their high insecticidal activity. Biosensors are potentially useful as they detected pesticides quickly and have been active in the research area for some years. Another valuable HRP-based biosensor was developed in which polyvinyl pyrrolidone (PVP) nanofibers were spun with incorporation of the enzyme HRP. Scanning electron microscopy (SEM) of the spun nanofibers was used to confirm the non woven structure which had an average diameter of 155 ± 34 nm. The HRP containing fibers were tested for their change in activity following electrospinning and during storage. A colorimetric assay was used to characterize the activity of HRP by reaction with the nanofiber mats in a microtiter plate and monitoring the change in absorption over time [63]. Rapid and sensitive detection methods are of utmost importance for the identification of pathogens related to health and safety. Peroxidase used in development of a nucleic acid sequence-based lateral flow assay which achieves a low limit of detection using chemiluminescence and enzymatic signal amplification [64].

### 4.10. Use in Pulp-Paper Industries

The pulping byproducts (black liquor) and pulp mill waste water cause serious environmental problem due to its high pollution load. Solving the pulp and paper industries' environmental problems is essential to maintaining the forest industry and accommodating the changing economic needs of forest communities [65]. Pulp manufacturing in pulp paper industries involve two main processes, that is, wood digestion and bleaching. In the process of wood digestion, wood chips are cooked in the solution of sodium hydroxide and sodium sulphate at elevated temperature and pressure to break chips into fiber mass. The chemical reaction with wood fibers dissolves all the depository materials which are hard to degrade, and these derivatives are washed away from the fiber during washing and dewatering process. Various extracts during washing include mainly lignins, cellulose, phenolics, resins, fatty acids, and tannins mixed together to make a dark black viscous alkaline waste known as black liquor. The alkaline effluent consists only in 10%–15% of total waste water but contributes in almost above 90%–95% of the total pollution load in terms of high pH, BOD, COD, and color which makes it significantly toxic to the environment [66]. Hence, the adequate treatment of black liquor prior to its discharge into environment is warranted. The biological methods for the treatment of black liquor involving the use of fungi, bacteria, algae, and enzymes as single step treatment or in combination with other physical and chemical methods seem to be more economical and ecofriendly [4]. Among biological methods tried so far, most of the literature is confined to a few genera of white rot fungi because of their nonspecific extracellular enzymatic system (LiP, MnP, and Laccase) involved in lignin biodegradation [67].

## 5. Conclusion

Importance of peroxidase in the detoxification of polluted environments relies on their capability to catalyse the reduction of peroxides, such as hydrogen peroxide and the oxidation of a variety of organic and inorganic compounds and the polymerization of toxic compounds or, by cross reaction, with other phenolics or with cosubstrates with toxic and harmless characteristics and generates polymeric products (dimmers, trimmers, hybrid oligomers), which will very likely accumulate in soil and/or in water systems. Peroxidases have potential for bioremediation of waste water contaminated with phenols, cresols, and other industrial effluents, for decolourization of textile dyes, removal of endocrine disruptive chemicals, degradation of pesticides, polychlorinated biphenyls, chlorinated alkanes and alkenes of soil, phenoxy alkanoic herbicides, triazine herbicides, chlorinated dioxins, and chlorinated insecticides. Peroxidases are also used as biosensors. Rapid progresses in the use of peroxidase for the degradation of pollutants have thrown more light on sustainable bioremediation strategies for polluting compounds and environmental protection by using different enzymes. Environmental protection is influenced by three interwoven factors: environmental legislation, ethics, and education. Each of these factors plays an important role in influencing national-level environmental decisions and personal-level environmental values and behaviors. For environmental protection to become a reality, it is important for societies and the nations to develop each of these areas that together will inform and drive environmental decisions.

## Figures and Tables

**Table 1:** Decolorization and detoxification of synthetic, textile dyes, and other industry effluent by microbial peroxidase(s).

S. No.	Type of peroxidase	Type of microorganism	Microorganism	Application	Reference
(1)	Peroxidase	Bacteria	*E*. *coli *	Dye degradation	[20]
(2)	Peroxidase	Bacteria	*Bacillus *sp. F31	Dye degradation	[21]
(3)	Manganese-dependent peroxidase (MnP), lignin peroxidase (LiP),	Fungi	Fourbasidiomycetous fungi (*Pleurotus ostreatus*sensu Cooke, *Coriolus versicolor* (L.) Quel., *Tyromyces albidus* (Schaeff.) Donk, and *Trametes gallica *	Biodelignification	[22]
(4)	Lignin peroxidase	Bacteria	*Citrobacterfreundii* (FJ581026) and *Citrobacter *sp. (FJ581023)	Black liquor (pulping by product cause serious environmental problem)	[4]
(5)	Lignin peroxidase	Yeast	*Candida krusei *	Basic Violet 3 (BV) extensively used in human and veterinary medicine as a biological stain and in various commercial textile processes	[23]
(6)	Lignin peroxidase	Bacterium	*Pseudomonas desmolyticum *	Diazo dye Direct Blue-6	[24]
(7)	Mn-peroxidase,	Bacterium	*Pseudomonas* sp.	Malachite green, a widely-used recalcitrant dye has been confirmed to be carcinogenic and mutagenic against many organisms.	[25]
(8)	Lignin peroxidase	White rot fungi	*Pleurotusostreatus *	Remazol Brilliant Blue R (Artificial dye)	[26]
(9)	Peroxidase	Bacterium	*Pseudomonas* sp*. *	Congo red decolorization	[27]
(10)	Lignin peroxidase isoenzymes (LiP 4.65, LiP 4.15, and LiP 3.85)	Fungus	*Phanerochaete chrysosporium *	Azo, triphenyl methane, heterocyclic, and polymeric dyes	[28]
(11)	Peroxidase	bacterium	*Clostridium bifermentans *	Reactive azo dyes	[29]
(12)	Versatile peroxidase	Fungus	*Thanatephorus cucumeris *	Anthraquinone dye Reactiveblue 5	[30]
(13)	DyP-type peroxidases	Fungi	*Auricularia auricula-judae *	High-redox potential dyes	[31]
(14)	Extracellular LiP	Bacteria	*Bacillus *sp*. *	Navy blue 2GL-azo dye	[15]
(15)	Dye-decolorizing peroxidases (DyP)	Fungi	*Pleurotusostreatus *	Azo dyes	[32]

## References

[1] Marco-Urrea E, Aranda E, Caminal G, Guillén F (2009). Induction of hydroxyl radical production in *Trametes versicolor* to degrade recalcitrant chlorinated hydrocarbons. *Bioresource Technology*.

[2] Hamid H, Rehman KU (2009). Potential applications of peroxidases. *Food Chemistry*.

[3] Chanwun T, Muhamad N, Chirapongsatonkul N, Churngchow N (2013). *Hevea brasiliensis* cell suspension peroxidase: purification, characterization and application for dye decolorization. *AMB Express*.

[4] Chandra R, Abhishek A, Sankhwar M (2011). Bacterial decolorization and detoxification of black liquor from rayon grade pulp manufacturing paper industry and detection of their metabolic products. *Bioresource Technology*.

[5] Huber P, Carré B (2012). Decolorization of process waters in deinking mills and similar applications: a review. *BioResources*.

[6] Lundell TK, Mäkelä MR, Hildén K (2010). Lignin-modifying enzymes in filamentous basidiomycetes-ecological, functional and phylogenetic review. *Journal of Basic Microbiology*.

[7] Hofrichter M, Ullrich R, Pecyna MJ, Liers C, Lundell T (2010). New and classic families of secreted fungal heme peroxidases. *Applied Microbiology and Biotechnology*.

[8] Marco-Urrea E, Reddy CA (2012). Degradation of chloro-organic pollutants by white rot fungi microbial degradation of xenobiotics. *Environmental Science and Engineering*.

[9] Ong ST, Keng PS, Lee WN, Ha ST, Hung WT (2011). Dye waste treatment. *Water*.

[10] Gopi V, Upgade A, Soundararajan N (2012). Bioremediation potential of individual and consortium non-adaptedfungal strains on Azo dye containing textile effluent. *Advances in Applied Science Research*.

[11] Tien HT, Salamon Z, Kutnik J (1988). Bilayer Lipid Membranes (BLM): an experimental system for biomolecular electronic device development. *Journal of Molecular Electronics*.

[12] Kirby N, McMullan G, Marchant R (1995). Decolourisation of an artificial textile effluent by *Phanerochaete chrysosporium*. *Biotechnology Letters*.

[13] Ng TW, Cai Q, Wong C, Chow AT, Wong P (2010). Simultaneous chromate reduction and azo dye decolourization by *Brevibacterium casei*: Azo dye as electron donor for chromate reduction. *Journal of Hazardous Materials*.

[14] Ghasemi F, Tabandeh F, Bambai B, Sambasiva Rao KRS (2010). Decolorization of different azo dyes by *Phanerochaete chrysosporium* RP78 under optimal condition. *International Journal of Environmental Science and Technology*.

[15] Dawkar VV, Jadhav UU, Jadhav SU, Govindwar SP (2008). Biodegradation of disperse textile dye Brown 3REL by newly isolated *Bacillus* sp. VUS. *Journal of Applied Microbiology*.

[16] Krishnaveni M, Kowsalya R (2011). Characterization and decolorization of dye and textile effluent by laccase from *Pleurotus florida*- A white-rot fungi. *International Journal of Pharma and Bio Sciences*.

[17] Pomar F, Caballero N, Pedreo MA, Ros Barceló A (2002). H_2_O_2_ generation during the auto-oxidation of coniferyl alcohol drives the oxidase activity of a highly conserved class III peroxidase involved in lignin biosynthesis. *FEBS Letters*.

[18] Shin JS, Kim BG (1997). Kinetic resolution of *α*-methylbenzylamine with *ω*-transaminase screened from soil microorganisms: application of a biphasic system to overcome product inhibition. *Biotechnology and Bioengineering*.

[19] Bhunia A, Durani S, Wangikar P (2002). Horseradish peroxidase catalyzed degradation of industrially important dyes. *Journal of Biotechnology and Bioengineering*.

[20] Gennaro PD, Bargna A, Bruno F, Sello G (2013). Purification of recombinant catalase-peroxidase HPI from *E.coli* and its application in enzymatic polymerization reactions. *Joural of Applied Microbiology and Biotechnology*.

[21] Bansal N, Kanwar SS (2013). Purification and characterization of an extracellular peroxidase of a bacterial isolate *Bacillus* sp. F31. *Current Biotechnology*.

[22] Hong Y, Dashtban M, Chen S, Song R, Qin W (2012). Enzyme production and lignin degradation by four basidiomycetousfungi in submerged fermentation of peat. *International Journal of Biology Medium*.

[23] Deivasigamani C, Das N (2011). Biodegradation of Basic Violet 3 by *Candida krusei* isolated from textile wastewater. *Biodegradation*.

[24] Kalme SD, Parshetti GK, Jadhav SU, Govindwar SP (2007). Biodegradation of benzidine based dye Direct Blue-6 by *Pseudomonas* desmolyticum NCIM 2112. *Bioresource Technology*.

[25] Du LN, Wang S, Li G (2011). Biodegradation of malachite green by *Pseudomonas* sp. strain DY1 under aerobic condition: characteristics, degradation products, enzyme analysis and phytotoxicity. *Ecotoxicology*.

[26] Shin KS, Young HK, Lim JS (2005). Purification and characterization of manganese peroxidase of the white-rot fungus *Irpex lacteus*. *Journal of Microbiology*.

[27] Telke AA, Joshi SM, Jadhav SU, Tamboli DP, Govindwar SP (2010). Decolorization and detoxification of Congo red and textile industry effluent by an isolated bacterium *Pseudomonas* sp. SU-EBT. *Biodegradation*.

[28] Ollikka P, Alhonmaki K, Leppanen V-M, Glumoff T, Raijola T, Suominen I (1993). Decolorization of azo, triphenyl methane, heterocyclic, and polymeric dyes by lignin peroxidase isoenzymes from *Phanerochaete chrysosporium*. *Applied and Environmental Microbiology*.

[29] Joe MH, Lim SY, Kim DH, Lee IN (2008). Decolorization of reactive dyes by *Clostridium bifermentans* SL186 isolated from contaminated soil. *World Journal of Microbiology and Biotechnology*.

[30] Sugano Y, Matsushima Y, Shoda M (2006). Complete decolorization of the anthraquinone dye Reactive blue 5 by the concerted action of two peroxidases from *Thanatephorus cucumeris*. *Applied Microbiology and Biotechnology*.

[31] Liers C, Bobeth C, Pecyna M, Ullrich R, Hofrichter M (2010). DyP-like peroxidases of the jelly fungus *Auricularia auricula*-judae oxidize nonphenolic lignin model compounds and high-redox potential dyes. *Applied Microbiology and Biotechnology*.

[32] Faraco V, Piscitelli A, Sannia G, Giardina P (2007). Identification of a new member of the dye-decolorizing peroxidase family from Pleurotus ostreatus. *World Journal of Microbiology and Biotechnology*.

[33] Gómez-Toribio V, García-Martín AB, Martínez MJ, Martínez ÁT, Guillén F (2009). Enhancing the production of hydroxyl radicals by Pleurotus eryngii via quinone redox cycling for pollutant removal. *Applied and Environmental Microbiology*.

[34] Kaušpediene D, Kazlauskiene E, Gefeniene A, Binkiene R (2010). Comparison of the efficiency of activated carbon and neutral polymeric adsorbent in removal of chromium complex dye from aqueous solutions. *Journal of Hazardous Materials*.

[35] Mui ELK, Cheung WH, Valix M, McKay G (2010). Dye adsorption onto activated carbons from tyre rubber waste using surface coverage analysis. *Journal of Colloid and Interface Science*.

[36] Longoria A, Tinoco R, Vázquez-Duhalt R (2008). Chloroperoxidase-mediated transformation of highly halogenated monoaromatic compounds. *Chemosphere*.

[37] Hakala TK, Hildén K, Maijala P, Olsson C, Hatakka A (2006). Differential regulation of manganese peroxidases and characterization of two variable MnP encoding genes in the white-rot fungus Physisporinus rivulosus. *Applied Microbiology and Biotechnology*.

[38] Mamatha J, Vedamurthy AB, Shruthi SD (2012). Degradation of phenol by turnip root enzyme extract. *Journal of Microbiology and Biotechnology Research*.

[39] Ruiz-Dueñas FJ, Morales M, García E, Miki Y, Martínez MJ, Martínez AT (2009). Substrate oxidation sites in versatile peroxidase and other basidiomycete peroxidases. *Journal of Experimental Botany*.

[40] Mossallam KF, Sultanova FM, Salemova NA Peroxidase catalysed the removal of phenol of phenol) from synthetic waste water.

[41] Zheng W, Colosi LM (2011). Peroxidase-mediated removal of endocrine disrupting compound mixtures from water. *Chemosphere*.

[42] Cabana H, Jones JP, Agathos SN (2007). Elimination of endocrine disrupting chemicals using white rot fungi and their lignin modifying enzymes: a review. *Engineering in Life Sciences*.

[43] Huang Q, Weber WJ (2005). Transformation and removal of bisphenol A from aqueous phase via peroxidase-mediated oxidative coupling reactions: efficacy, products, and pathways. *Environmental Science and Technology*.

[44] Renner G (1980). Metabolic studies on pentachloronitrobenzene (PCNB) in rats. *Xenobiotica*.

[45] McCauley LA, Anger WK, Keifer M, Langley R, Robson MG, Rohlman D (2006). Studying health outcomes in farmworker populations exposed to pesticides. *Environmental Health Perspectives*.

[46] Raymond J, Rogers T, Shonnard D, Kline A (2001). A review of structure-based biodegradation estimation methods. *Journal of Hazardous Materials*.

[47] Jauregui J, Valderrama B, Albores A, Vazquez-Duhalt R (2003). Microsomal transformation of organophosphorus pesticides by white rot fungi. *Biodegradation*.

[48] Harayama S (1997). Polycyclic aromatic hydrocarbon bioremediation design. *Current Opinion in Biotechnology*.

[49] Weber R, Gaus C, Tysklind M (2008). Dioxin- and POP-contaminated sites-contemporary and future relevance and challenges: overview on background, aims and scope of the series. *Environmental Science and Pollution Research*.

[50] Harford-Cross CF, Carmichael AB, Allan FK, England PA, Rouch DA, Wong L (2000). Protein engineering of cytochrome P458(cam) (CYP101) for the oxidation of polycyclic aromatic hydrocarbons. *Protein Engineering*.

[51] Kordon K, Mikolasch A, Schauer F (2010). Oxidative dehalogenation of chlorinated hydroxybiphenyls by laccases of white-rot fungi. *International Biodeterioration and Biodegradation*.

[52] Yadav JS, Bethea C, Reddy CA (2000). Mineralization of trichloroethylene (TCE) by the white rot fungus *Phanerochaete chrysosporium*. *Bulletin of Environmental Contamination and Toxicology*.

[53] Huang X, Pan J, Liang B, Sun J, Zhao Y, Li S (2009). Isolation, characterization of a strain capable of degrading imazethapyr and its use in degradation of the herbicide in soil. *Current Microbiology*.

[54] Bending GD, Friloux M, Walker A (2002). Degradation of contrasting pesticides by white rot fungi and its relationship with ligninolytic potential. *FEMS Microbiology Letters*.

[55] Kasai N, Ikushiro S, Shinkyo R (2010). Metabolism of mono- and dichloro-dibenzo-p-dioxins by *Phanerochaete chrysosporium* cytochromes P450. *Applied Microbiology and Biotechnology*.

[56] Quintero JC, Moreira MT, Feijoo G, Lema JM (2008). Screening of white rot fungal species for their capacity to degrade lindane and other isomers of hexachlorocyclohexane (HCH). *Ciencia e Investigacion Agraria*.

[57] Mougin C, Pericaud C, Malosse C, Laugero C, Asther C (1996). Biotransformation of the insecticide lindane by the white rots basidiomycete *Phanerochaete chrysosporium*. *Journal of Pest Science*.

[58] Purnomo AS, Mori T, Kamei I, Nishii T, Kondo R (2010). Application of mushroom waste medium from *Pleurotus ostreatus* for bioremediation of DDT-contaminated soil. *International Biodeterioration and Biodegradation*.

[59] Ahammad S (2013). Hydrogen peroxide biosensors based on horseradish peroxidase and haemoglobin. *Jounal of Biosensors & Bioelectronics*.

[60] Song J, Xu J, Zhao P, Lu L, Bao J (2011). A hydrogen peroxide biosensor based on direct electron transfer from hemoglobin to an electrode modified with Nafion and activated nanocarbon. *Microchimica Acta*.

[61] Xu S, Zhang X, Wan T, Zhang C (2011). A third-generation hydrogen peroxide biosensor based on horseradish peroxidase cross-linked to multi-wall carbon nanotubes. *Microchimica Acta*.

[62] Qiu J, Cui S, Liang R (2010). Hydrogen peroxide biosensor based on the direct electrochemistry of myoglobin immobilized on ceria nanoparticles coated with multiwalled carbon nanotubesby a hydrothermal synthetic method. *Microchimica Acta*.

[63] Dai M, Jin S, Nugen SR (2012). Water-soluble electrospunnano fibers as a method for on-chip reagent storage. *Biosensors*.

[64] Wang Y, Fill C, Nugen SR (2012). Development of chemiluminiscent lateral flow assay for the detection of nucleic acids. *Biosensors*.

[65] Khalid A, Arshad M, Crowley DE (2009). Biodegradation potential of pure and mixed bacterial cultures for removal of 4-nitroaniline from textile dye wastewater. *Water Research*.

[66] Grover R, Marwaha SS, Kennedy JF (1999). Studies on the use of an anaerobic baffled reactor for the continuous anaerobic digestion of pulp and paper mill black liquors. *Process Biochemistry*.

[67] Hammel KE, Cullen D (2008). Role of fungal peroxidases in biological ligninolysis. *Current Opinion in Plant Biology*.

